# *Drosophila* Hox and Sex-Determination Genes Control Segment Elimination through EGFR and *extramacrochetae* Activity

**DOI:** 10.1371/journal.pgen.1002874

**Published:** 2012-08-09

**Authors:** David Foronda, Paloma Martín, Ernesto Sánchez-Herrero

**Affiliations:** Centro de Biología Molecular Severo Ochoa (C.S.I.C.-U.A.M.), Universidad Autónoma de Madrid, Cantoblanco, Madrid, Spain; University of California San Francisco, United States of America

## Abstract

The formation or suppression of particular structures is a major change occurring in development and evolution. One example of such change is the absence of the seventh abdominal segment (A7) in *Drosophila* males. We show here that there is a down-regulation of EGFR activity and fewer histoblasts in the male A7 in early pupae. If this activity is elevated, cell number increases and a small segment develops in the adult. At later pupal stages, the remaining precursors of the A7 are extruded under the epithelium. This extrusion requires the up-regulation of the HLH protein Extramacrochetae and correlates with high levels of *spaghetti-squash*, the gene encoding the regulatory light chain of the non-muscle myosin II. The Hox gene *Abdominal-B* controls both the down-regulation of *spitz*, a ligand of the EGFR pathway, and the up-regulation of *extramacrochetae*, and also regulates the transcription of the sex-determining gene *doublesex*. The male Doublesex protein, in turn, controls *extramacrochetae* and *spaghetti-squash* expression. In females, the EGFR pathway is also down-regulated in the A7 but *extramacrochetae* and *spaghetti-squash* are not up-regulated and extrusion of precursor cells is almost absent. Our results show the complex orchestration of cellular and genetic events that lead to this important sexually dimorphic character change.

## Author Summary

Many species display sexually dimorphic characters in specific regions of their body. In *Drosophila melanogaster*, a striking difference between males and females is the development of the seventh abdominal segment (A7), absent in males. We have found that in the first 30 h of pupal development, proliferation in the male A7 is reduced as compared to that of other abdominal segments, resulting in a small primordium. The *Epidermal growth factor receptor* pathway, which is in part responsible for this reduction, is down-regulated in male A7 cells, and if the activity of the pathway is increased there is a small seventh segment in the adult male. In later stages of pupal development, the remaining cells of the male A7 invaginate and die, and this requires the activity of myosin regulated by the gene *extramacrochetae*. Extramacrochetae levels of expression are increased in the male, but not female, A7 cells, suggesting that the sex determination pathway regulates this sexual difference (absence or not of the A7) by governing this gene. The Hox gene *Abdominal-B*, required to specify the posterior abdominal segments, controls both down-regulation of the *Epidermal growth factor receptor* pathway and extrusion, the latter partly through the regulation of the transcription of *doublesex*, a key gene in the sex determination pathway.

## Introduction

A major change during evolution is the disappearance of a particular organ or structure. This event is sometimes restricted to one sex, and therefore needs the coordination between sex-determination genes and those that control pattern [1], such as the Hox genes, a group of genes that specify different structures along the antero-posterior axis [2]. One example of such coordination is the control of pigmentation in the *Drosophila melanogaster* posterior abdomen, uniformly pigmented in males but not in females. This character depends on the Hox gene *Abdominal-B* (*Abd-B*), required in abdominal (A) segments A5-A9 [3]–[5], and whose protein levels increase gradually and posteriorwards in each segment [6], [7], and also on the two protein isoforms of the sex-determining gene *doublesex* (*dsx*): DsxM (in males) and DsxF (in females). The combined activity of these proteins and *Abd-B* promotes the development of sex-specific pigmentation [8], [9].

Another significant morphological difference between *Drosophila* males and females is the seventh abdominal segment (A7), absent in males and present in females. Abdominal segments derive from histoblast nests, groups of cells that intermingle with cuticular larval epidermal cells (LECs), are quiescent during the larval period and proliferate rapidly at the beginning of the pupal period [10]–[13]. There are four histoblast nests in each hemi-segment: two dorsal (anterior, a, which forms the dorsal part of the abdominal cuticle, the tergite, and posterior, p), one ventral, developing the ventral region (the sternite) and part of the lateral region (the pleura), and one making the spiracle [12], [13]. When pupation starts, the histoblasts proliferate and spread, whereas the larval epidermal cells that are contiguous to them die and are extruded, until the whole abdominal region is covered by the histoblasts, which secrete the adult cuticle [11]–[14].

The study of the elimination of the male A7 has been recently addressed [15]. In this analysis, it was demonstrated that the absence of *wingless* (*wg*) expression in the male A7 segment contributes to the disappearance of this metamere, probably by regulating cell proliferation. It was also shown that the forced expression of a ligand of the Epidermal growth factor receptor (EGFR) pathway, *vein*, makes a small A7 in the male and that segment compartmental transformation (from A7p to A6a) and restricted apoptosis also contribute to the sexual dimorphism in this segment.

We have studied the mechanisms of male A7 elimination and report here that at early pupa there is less number of histoblasts in the male A7 due, at least in part, to the down-regulation by *Abd-B* of the activity of the EGFR pathway; at later pupal stages the A7 histoblasts undergo extrusion under the control of the HLH protein Extramacrochetae (Emc). *Abd-B* regulates *dsx* expression in males and females, but only DsxM drives the massive extrusion of male A7. Our results show that different cellular events, under the joint regulation of the sex-determination pathway and Hox activity, underlie the disappearance of a particular structure.

## Results

The different development of the *Drosophila* A7 in males and females (Figure S1A, B) depends on *Abd-B* [3], [4] and on the sex-determination pathway [16] (Figure S1C, D). We have studied the elimination of the male A7 tergite by comparing the behavior of dorsal histoblasts in the A6 (which remains) and the A7 (which disappears). The expression driven by a *escargot* (*esg*)-Gal4 line [17] in the abdomen marks specifically the histoblasts, which can be also distinguished from surrounding LECs because they are diploid (and small) whereas the LECs are polytenic (and big). To permanently label histoblasts we have used a genetic combination that we name (*p*)*esg*-Gal4 [12] (see Materials and Methods].

### EGFR pathway activity is reduced in the male A7

At the end of the larval stages the number of histoblasts in the male A7a and A7p dorsal nests is similar to the corresponding nests of the A6 ([11]; and data not shown). In the first 10h after puparium formation (APF) the histoblasts undergo three nearly-synchronic divisions without cell growth, thus defining the first phase of histoblast pupal development [12], [13], [18], [19]. Time-lapse movies show that during this phase the A6a and A7a nests, which develop into the A6 and A7 tergites, respectively, show similar cell division rates, with just a small delay in the A7a nest.

**Figure 1:**
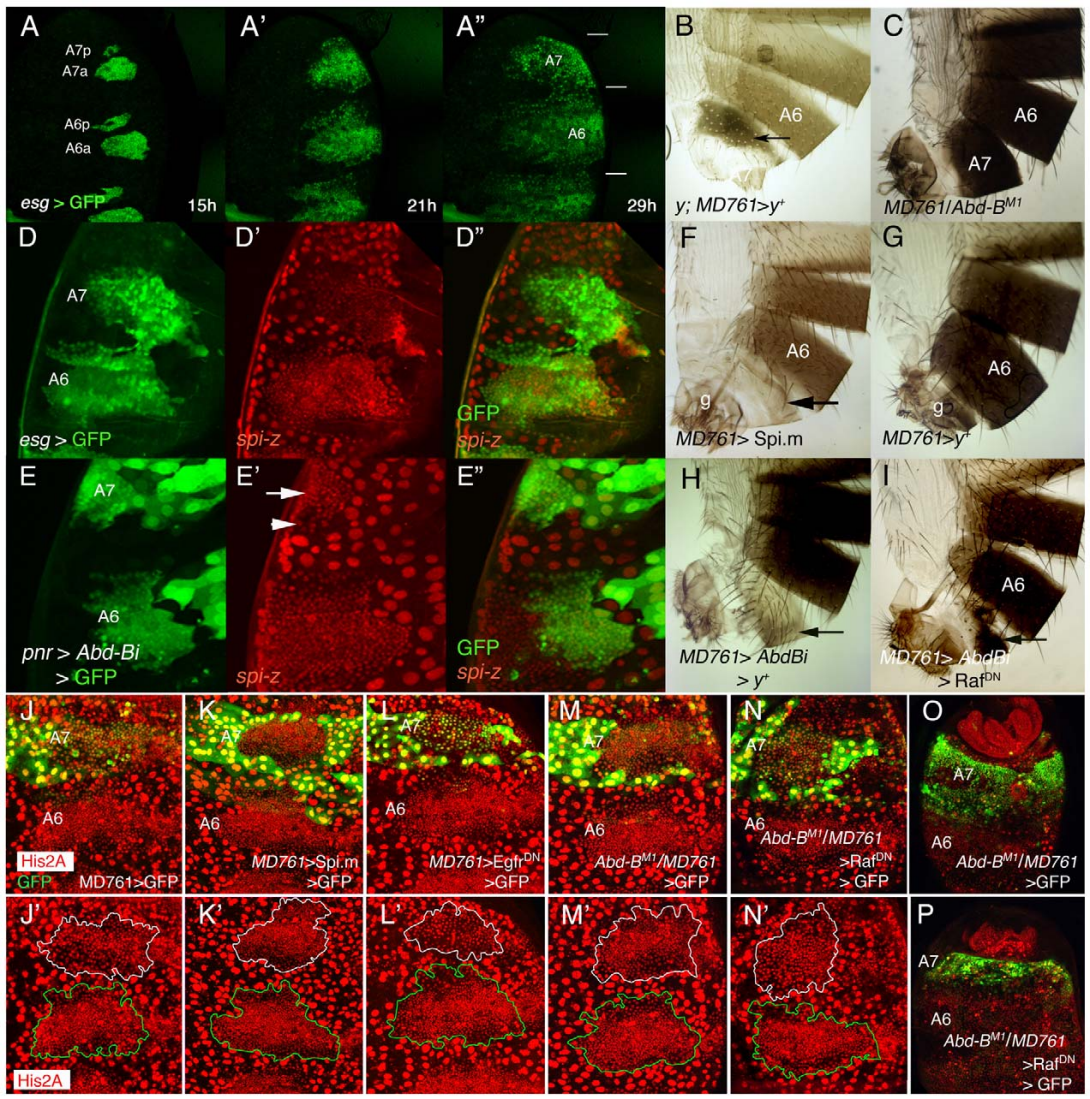
Cell divisions and EGFR activity in the male A7. (A–A″) Snapshots from Video S1 (*esg*-Gal4 UAS-*nls-myc*-GFP pupa), from ∼15 to 29 h APF showing the expansion of the dorsal histoblast nests in the male A6 and A7 segments. In this and subsequent figures (except in adult cuticle preparations) the posterior part of the pupa is at the top. Note that the size of the A7 is smaller than that of the A6 (horizontal bars in A″). Approximate hours of development APF are also indicated. (B) *y*; *MD761*-Gal4 UAS-*y^+^*/+ female. Note the dark pigmentation in the A7 (arrow). (C) *MD761*-Gal4/*Abd-B^M1^* male. See that the A7 (absent in the wildtype) is almost completely transformed into the A6. (D–D″) Posterior part of a *spi*-lacZ *esg*-Gal4 UASGFP pupa of about 26 h APF showing that *spi* levels (in red) are reduced in the A7 histoblasts (the A7/A6 ratio of signal intensity is 0,82±0,12; n = 10). Histoblasts are marked by *esg* in green. (E–E″) If we express a UAS-*Abd-BRNAi* (UAS-*Abd-Bi*) construct under the control of the *pannier* (*pnr*)-Gal4 driver (domain of expression in green), the levels of *spi*-lacZ are elevated in this domain (arrow). The arrowhead marks the lower *spi*-lacZ levels in A7 histoblasts where *Abd-B* expression is still high (note also the bigger cells). (F) UAS-Spi.m-GFP; *MD761*-Gal4/+ adult male (cross made at 17°C), with a small A7 segment (compare with a male expressing a UAS-*y+* construct (G), which, like the wildtype, has no A7); g, genitalia. (H) UAS-*Abd-BRNAi*/+; *MD761*-Gal4 UAS-*y^+^*/+adult male, showing the transformation of the A7 into the A4. (I) In UAS-*Abd-BRNAi*/+; *MD761*-Gal4/UAS-Raf^DN^ the size of the A7 segment is reduced as compared to that shown in H. (J–L′). Posterior abdomens of ∼22–24 h APF male pupae of the following genotypes: *His2A-RFP*/+; *MD761*-Gal4 UAS-GFP/+ (J, J′), UAS-Spi.m-GFP; *His2A-RFP*/+; *MD761*-Gal4 UAS-GFP/+ (K, K′), and *His2A-RFP*/UAS-Egfr^DN^; *MD761*-Gal4 UAS-GFP/+ (L, L′), showing a slight reduction (L, L′) and an increase (K, K′) in histoblast number in the A7 with respect to the *His2A-RFP*/+; *MD761*-Gal4 UAS-GFP/+ pupae (J, J′): at about this time, in the wildtype, the histoblast number A7/A6 ratio is 0’47±0’07 (n = 4), it is 0’95±0’19 (n = 4) when we express *mspi* in the A7, and 0’31±0’04 (n = 4) when the Raf^DN^ product is present in the same segment. (M, M′) In *MD761*-Gal4 UAS-GFP/*Abd-B^M1^* male pupa the number of histoblasts in the A7a dorsal nests approaches that of the A6a, and this number is reduced in *MD761*-Gal4 UAS-GFP/UAS-Raf^DN^ *Abd-B^M1^* pupae (N, N′). At later stages, the A7 of the *MD761*-Gal4 UAS-GFP/*Abd-B^M1^* pupae (O; marked in green) is bigger than the wildtype and it is strongly reduced when a Raf^DN^ protein is concomitantly expressed in this genetic background (P). Nuclei are marked in red and the A7 delimited by GFP expression (in green). In the lower panels (J′, K′, L′, M′, N′) the A7 dorsal nests are delineated in white and the A6 ones in green.

In a second phase, from ∼10 h to ∼35 h APF, the histoblasts divide asynchronously and cell division is accompanied by cell growth [12], [13], [19]. Histoblasts also spread in the epidermis and replace LECs [12], [14]. During this phase the male A7 histoblasts undergo fewer cell divisions than those of the A6 ([15]; and our observations) so that their number at about 24–29 h APF is smaller than that of the A6 (Video S1; Figure 1A–A″, 1J, J′). We also note that the size of the A7 histoblasts is bigger than that of the A6 histoblasts (Figure 1J, J′; Figure S1E–E″).

*Abd-B* levels are higher in the pupal A7 than in the A6 [8] (Figure S1G, G′). By transforming the A6 into the A7 with the *Abd-B^Fab7-1^* mutation [20], [21], we observed a concomitant change in Abd-B levels, cell number and cell size (Figure S1G-H′). To study the reciprocal transformation we used a Gal4 line (*MD761*-Gal4) that is inserted within the *infraabdominal-7* (*iab-7*) region of this gene (position between 3R:12,725,043 and 3R:12,725,044, Flybase), close to or within the *Fab-7* boundary [20]–[22]. The *iab-7* regulatory domain activates *Abd-B* in parasegment 12 (A6p-A7a) [20], [21]. In accordance with its location, *MD761*-Gal4 drives expression of UAS constructs in this parasegment and some posterior cells (M. Calleja and G. Morata, personal communication; Figure 1B). In addition to being an enhancer trap that expresses Gal4 in PS12, the insertion disrupts regulatory sequences and results in a strong *iab-7* mutation that, when *in trans* to *Abd-B* null mutations, substantially reduces *Abd-B* expression in the A7, transforms this segment into the A6, and makes the A7 histoblast size and number resemble those of the A6 (Figure 1C; Figure S1F–G′). We conclude that changes in *AbdB* expression levels are necessary and sufficient to regulate the differences in cell size and number between the A6 and the A7.

The EGFR pathway regulates the second phase of histoblast development [19]. We have found that the expression of *spitz* (*spi*), a ligand of the EGFR pathway present both in histoblasts and LECs [19], and of *argos*, a target of the pathway [23], are reduced in the male A7 as compared to that of more anterior nests (Figure 1D–D″; Figure S1I, I′; the reduction is weakly detected in some cases). As expected, this different *spi* expression depends on *Abd-B* (Figure 1E–E″). The down-regulation of EGFR ligand expression seems to be important because forcing the expression of the unprocessed form of Spi (Spi.m) [24] (Figure 1F; Figure S1J), of an activated form of Ras (Ras^V12^) [25] (Fig, S1K), or of another EGFR ligand, *vein* [15], allows the formation of a reduced A7 segment (compare with a *MD761* UAS-*y^+^/+* male in Figure 1G). Further, the transformation of A7 into A4 observed in *MD761*-Gal4 UAS-*Abd-BRNAi* flies (Figure1H) is substantially reduced if we co-express a dominant negative form of the Epidermal growth factor receptor [26] (Figure S1L), a dominant negative form of Raf, a protein that transduces the signal [27], [28] (Figure 1I), or the wildtype Argos protein, which inhibits the pathway [23] (Figure S1M). We also observed in these mutants changes in histoblast cell number: thus, an increase or a reduction in activity of the EGFR pathway in the A7 augments or diminishes, respectively, histoblast number at about 22–24 h APF (Figure 1K, K′, L, L′, the wildtype in 1J, J′). In a similar way, if *Abd-B* expression is reduced, the number of A7 histoblasts increases to resemble that of the A6 (Figure 1M, M′), and this increase is partially reverted if the EGFR pathway is down-regulated (Figure 1N, N′). The difference A7 size in these two genotypes is observed later in development, after full expansion of the nests (Figure 1O, P). All these results suggest that high levels of *AbdB* down-regulate EGFR activity in the A7 and that this regulation probably impinges in the number of A7 cells and in A7 size after full histoblast expansion.

### Male A7 histoblasts are extruded through the epithelium and die

**Figure 2:**
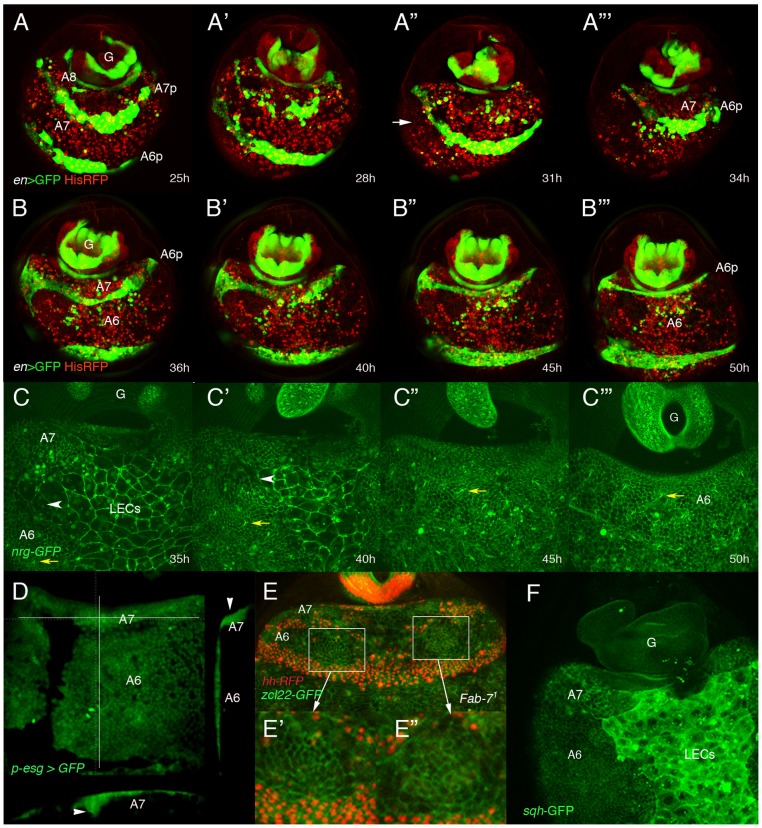
Dorsal histoblasts of the male A7 extrude through the epithelium. (A–B″′) Stills from Videos S2 and S3 showing the progressive elimination, first of A8 LECs and then of A7 LECs and histoblasts, in His2A-RFP/*en*-Gal4 UAS-GFP male pupae from about 25 h till about 50 h APF. En is expressed in posterior compartments, (marked in green by GFP), and nuclei are marked in red. In this and other movies (and snapshots from them) marked with His2A-RFP, histoblasts are difficult to see under the moving cells (macrophages or hemocytes), which present a strong red signal. A histoblast nest is indicated by an arrow in A″. A8 cells disappear first and A7 cells follow, so that A6p histoblasts end up contacting the genitalia (G), which rotates during this period. Numbers indicate approximate hours APF. (C–C″′) Snapshots from Video S5 (approx. 35–50 h APF) showing the apparent disappearance of A7 histoblasts in an *nrg*-GFP male pupa. The yellow arrows indicate the position of a bristle precursor and the white arrowheads shows the LECs separating the A6p and A7a histoblast nests. Note how both marks move posteriorly as the A7 histoblasts are eliminated. Numbers indicate approximate hours APF. (D) Snapshot from a movie showing a (*p*)*esg*-Gal4 UAS-GFP male pupa of about 40 h APF; cross sections, to the right and below (the plane of section indicated by white lines) show the accumulation of histoblasts as bulges under the epidermis (arrowheads). (E) Still taken from video S6, showing an *Abd-B^Fab7-1^* homozygous male pupa in which Hh-RFP marks posterior compartments (in red) and the membrane marker *zcl22*-GFP [30] is in green. E′, E″ are details of the squares in E, showing the constriction of cells in this optical section in two regions of the histoblast nests before histoblast invagination. (F) *sqh*-GFP expression is higher in the A7 histoblast nests of a ∼36 h APF male pupa than in the A6.

To study why the male A7 histoblasts, although reduced in number, do not form an adult A7 segment, we made time-lapse movies of the posterior abdomen marking posterior compartments with *en*-Gal4 and nuclei with His2A-RFP. Although it has been shown that some A7a histoblasts show *de novo en* expression in pupa [15], this change is unlikely to alter the general effects we have seen: at ∼25–35 hours APF, we observed the apparent progressive disappearance of the A8 segment, the one abutting the rotating genitalia (Video S2; Figure 2A–A″′). This is remarkable, as it suggests that all the LECs of this segment may be extruded without the help of histoblasts (absent in the A8), something that occurs only in a reduced number of LECs from other segments [29]. From ∼36 to ∼45 h APF we also observed a similar apparent and gradual elimination of the A7 field; as a result of this effect, the A6 cells seem to move backwards, until A6p cells contact with the genital disc (Video S3; Figure 2B–B″′). This is also observed with the (*p*)*esg*-Gal4 UAS-GFP and *neuroglian*-GFP (*nrg*-GFP) [30] markers (Videos S4 and S5; Figure 2C–C″′ and Figure S2A). Optical Z-sections of the A7 segment in the former movie show the accumulation of histoblasts underneath the epidermis (Figure 2D), indicating the A7 histoblasts, like the LECs, undergo delamination (see also below, Videos S9 and S10).

Because of the curvature of the pupal abdomen, a better resolution of the movement and extrusion is observed in *Abd-B^Fab7-1^* homozygous pupae, in which both the A6 and A7 invaginate. In these pupae we observed that the extrusion seemed to be concentrated in two wide regions of cells (left and right) close to the LECs, and where cells show in optical sections a reduced apical size (Video S6 and Figure 2E–E″). This suggests that, similarly to LECs [12], non-muscle myosin may be required for histoblast extrusion. Consistently, the levels of *spaghetti-squash*, encoding the regulatory light chain of the non-muscle myosin II [31], are elevated at ∼35–40 h APF in the male A7 as compared to the A6 (Figure 2F). Furthermore, expressing a constitutively active form of the myosin binding subunit (MbsN300), a subunit of the phosphatase that inhibits myosin activity [32], we delay extrusion of larval cells [12] and of histoblasts (Video S7 and Figure S2B; the wildtype in Video S8 and Figure S2C). Male adults of the *MD761*-Gal4 UAS-MbsN300 genotype present a small A7, unpigmented and without bristles (Figure S2E, compare with the wildtype in Figure S2D). Taken together, the data suggest that A7 histoblasts invaginate like LECs and that myosin II is required for this extrusion.

**Figure 3:**
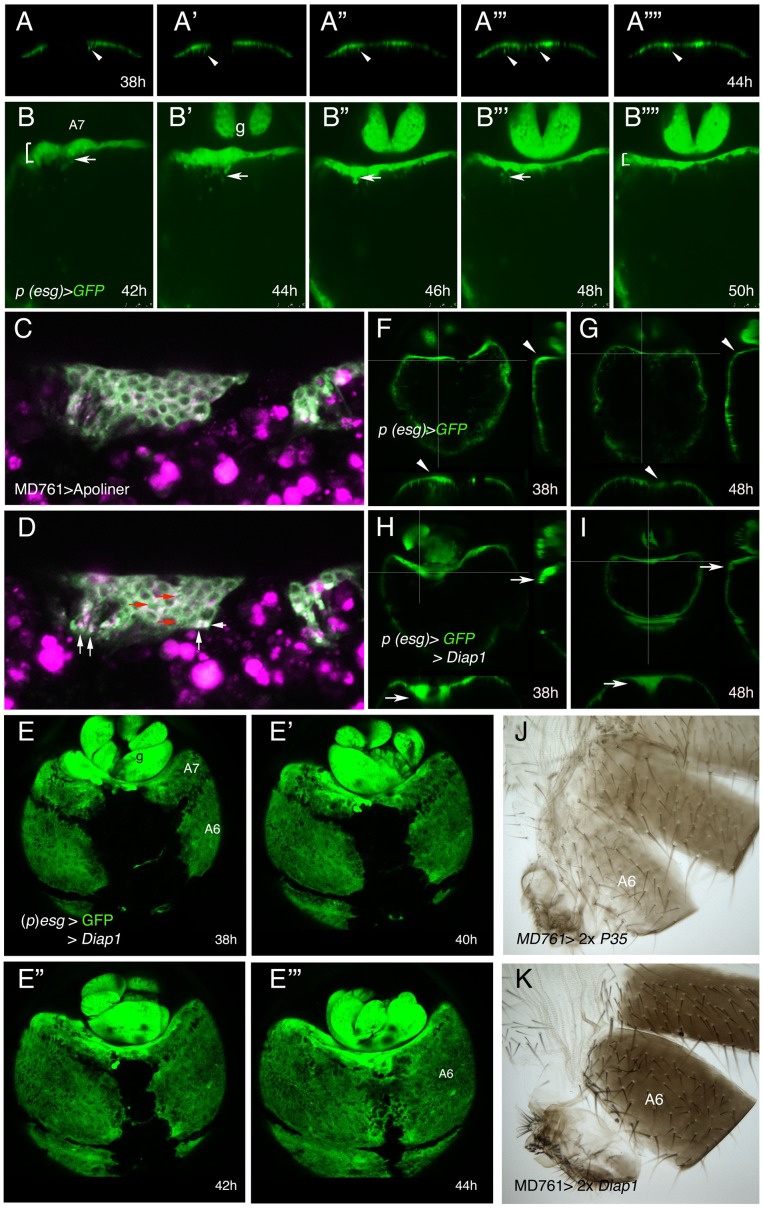
Inhibition of cell death does not prevent delamination of histoblasts. (A–A″″) Snapshots from video S9, from about 38 to 44 h APF, showing how the histoblasts from the male A7 left and right anterior dorsal nests of a (*p*)*esg*-Gal4 UAS-GFP male pupa delaminate as the nests meet at the central midline. The arrows indicate the delamination of some histoblasts. (B–B″″) Stills from video S10, made in a (*p*)*esg*-Gal4 UAS-GFP male pupa of about 42–50 h APF, showing delamination of A7 histoblasts (arrows). See how the “width” of the A7 segment (brackets at 42 h and 50 h) is reduced as delamination proceeds; g, genitalia. Numbers indicate approximate hours APF. (C, D) Two optical sections of a movie sequence in which the Apoliner construct, which reveals cell death [59], is expressed under the control of the *MD761*-Gal4 driver in A7 histoblasts. The panel C is from about 40 h APF and panel D from about 42 h APF. The red arrows indicate three cells where the GFP reporter has nuclear localization (indicating apoptosis) and the white arrows point to what could be apoptotic bodies. (E–E′″) Snapshots from video S11 showing the invagination of the male A7 in (*p*)*esg*-Gal4 UAS-GFP UAS-*Diap1* (*esg*-Gal4 *act*>*y^+^*>Gal4/UAS-*Diap1*; UAS-*flp*/UAS-*Diap1*) pupae. The invagination takes place as in the wildtype although it may be delayed. Numbers indicate approximate hours APF. (F–I) Optical sections of (*p*)*esg*-Gal4 UAS-GFP (F, G) and (*p*)*esg*-Gal4 UAS-*Diap1* UAS-GFP (H, I) male pupae. Cross-sections (white lines) to the right and below in each figure show that in (*p*)*esg*-Gal4 UAS-GFP pupae there are some histoblasts under the epithelium at about 38 h APF (F), but they are not longer there by 48 h APF (G) (arrowheads). By contrast, in (*p*)*esg*-Gal4 UAS-*Diap1* UAS-GFP male pupae, the number of A7 histoblasts that remain under the epithelium is higher at about 38 h APF (H) and have not been completely eliminated by 48 h APF (I) (arrows). (J, K) The inhibition of cell death in UAS-*P35*/+; UAS-*P35*/*MD761*-Gal4 (J) and UAS-*Diap1*/+; UAS-*Diap1*/*MD761*-Gal4 (K) males does not prevent A7 elimination.

The extrusion of LECs is accompanied by their death and clearance by macrophages [12], [29]. We also observed delamination (Videos S9 and S10; Figure 3A–A″″, B–B″″) and cell death (Figure 3C, D) of some histoblasts in the A7 dorsal nests. However, if we inhibit apoptosis by expressing the Diap1 protein, which prevents cell death [33], A7 histoblasts seem to be extruded (Video S11; Figure 3E–E′″), though their final elimination takes longer than in the wildtype (Figure 3F–I). However, cell death, although required for the efficient final elimination of histoblasts, is immaterial as to A7 suppression: the expression of cell death-inhibitors like P35 [34], *puckered* [35] or *Diap1* in the A7 does not prevent the disappearance of this segment [15] (Figure 3J, K and data not shown).

### The *extramacrochetae* gene is required for the extrusion of male A7 histoblasts

**Figure 4:**
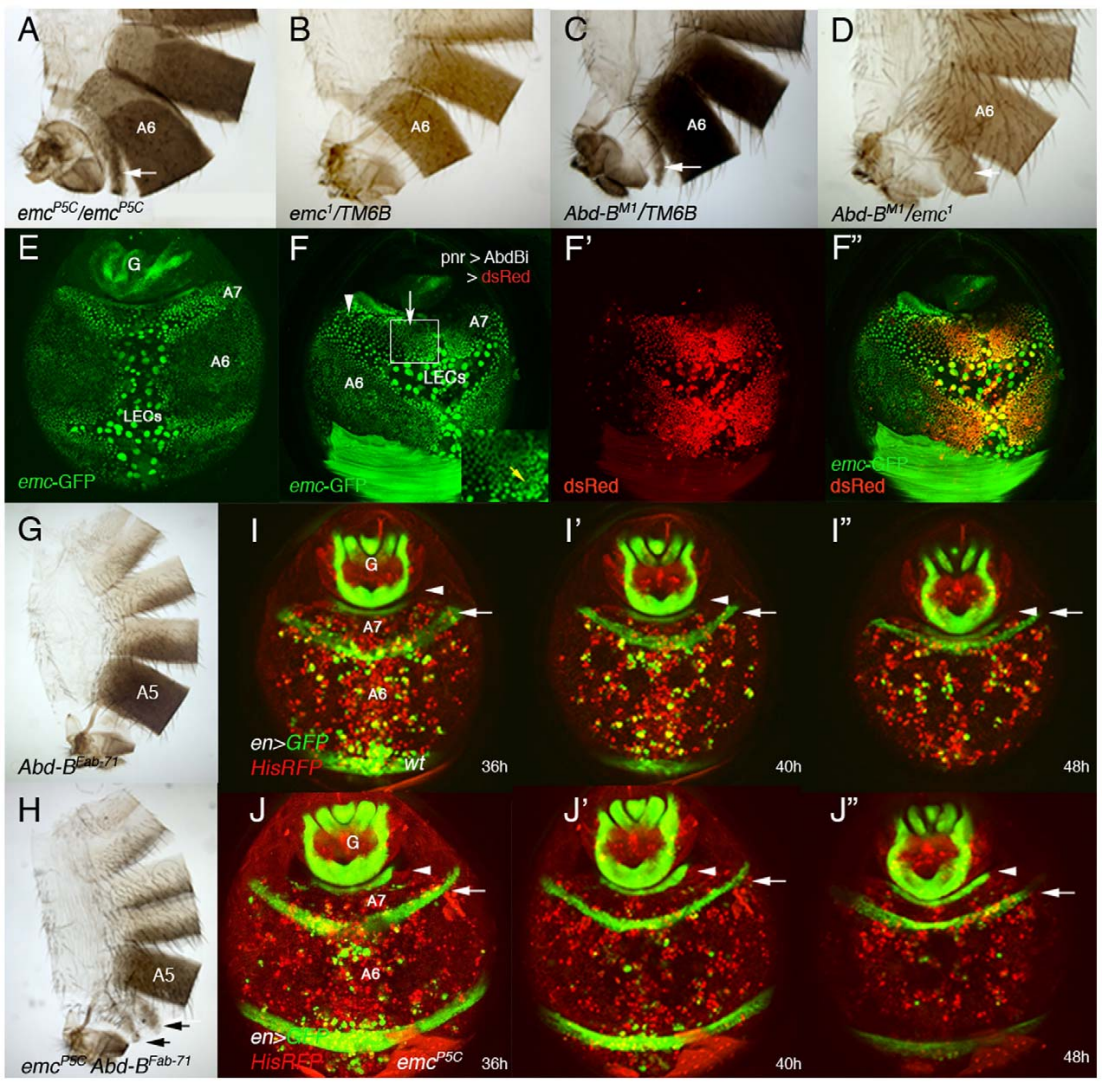
*emc* regulates extrusion of the male A7 histoblasts. (A, B) *emc^P5C^* homozygous males show a small A7 segment (A), absent in *emc^−^* heterozygous conditions (B). (C) Males heterozygous for an *Abd-B* mutation present a small A7 [3], [4], and the *trans*-heterozygous combination *Abd-B^M1^*/*emc^1^* enhances this phenotype (D). Arrows indicate the A7 segment. (E) Distribution of *emc*-GFP in the A7 and A6 segments of a ∼38 h APF male pupa. There is *emc* expression in LECs and histoblasts, with higher levels in A7 than in A6 histoblasts. We have measured the difference in signal intensity between nuclei of the two segments and found that the A7/A6 signal ratio is 1, 32±0,15 (n = 4). See also higher levels at the periphery of histoblast nests. (F–F″) UAS-DsRed/+; *emc*-GFP *pnr*-Gal4/UAS-*Abd-BRNAi* male pupa of about 36 h APF showing that in the central region of *pnr* expression (red in F′, F″), where *Abd-B* levels are reduced, *emc*-GFP levels are also reduced (arrow in F). Levels remain high where *Abd-B* has not been eliminated (arrowhead in F) but also in midline cells (also with high expression in anterior segments; yellow arrow in inset). (G) *Abd-B^Fab7-1^* homozygous adult male. The A6 disappears as it is transformed into the A7 [20]. (H) *Abd-B^Fab7-1^ emc^P5C^* homozygous male: there are small A6 and A7 segments (arrows), showing the *emc^P5C^* mutation is epistatic over the *Abd-B^Fab7-1^* mutation. (I–I″) Snapshots from Video S12 in a ∼36–48 h APF *en*-Gal4 UAS-GFP/His2A-RFP male, showing the progressive disappearance of the A7 segment. Posterior compartments show *en* expression (in green), whereas nuclei are labeled in red. The arrow marks the A6p band of expression and the arrowhead the A8p cells. (J–J″) Snapshots from Video S13, taken at similar stages and with the same markers but in an *emc* mutant background (*en*-Gal4 UAS-GFP/His2A-RFP; *emc^P5^*/*emc^P5^*male). Note that, contrary to the previous panels (I–I″), the A6p en band does not move posteriorly, indicating that the A7 segment is not being extruded. Numbers indicate approximate hours APF.

We have found that males with reduced function in the *extramacrocheate* (*emc*) gene, which encodes a HLH protein [36]–[38] with homology to vertebrate ID proteins, develop a small A7 segment (Figure 4A and Figure S3A–C). Different crosses among *Abd-B* and *emc* mutations reveal genetic interactions between these two genes in A7 development (Figure 4B–D; Figure S3D–M).

We studied *emc* expression with an *emc*-GFP enhancer trap [39] and found that *emc* is expressed both in LECs and histoblasts. Importantly, male pupae of about 36–42 h APF show an increase in *emc*-GFP expression in A7 dorsal histoblasts as compared with A6 ones (Figure 4E). As predicted, this higher expression depends on *Abd-B* levels (Figure 4F–F″). Consistently, *emc* mutations are epistatic over the *Abd-B^Fab7-1^* mutation (Figure 4G, H) and an increase in Emc can partially suppress the A7 segment produced by *Abd-B* mutations (Figure S3N, O). To ascertain the role of *emc* we made time-lapse movies in ∼36–48 h APF *emc^P5C^* male pupae and found that the extrusion of the dorsal A7 histoblasts is largely prevented (Video S13, and Figure 4J–J″, compare with the wild-type in Video S12 and Figure 4I–I″), although invagination of larval cells is not greatly disturbed. A similar result is observed in other *emc* mutant combinations, although a strong reduction in *emc* levels also affect LECs extrusion (not shown). Collectively, these results strongly suggest that *Abd-B* promotes suppression of male A7, at least in part, by regulating histoblast extrusion through the control of *emc*.

**Figure 5:**
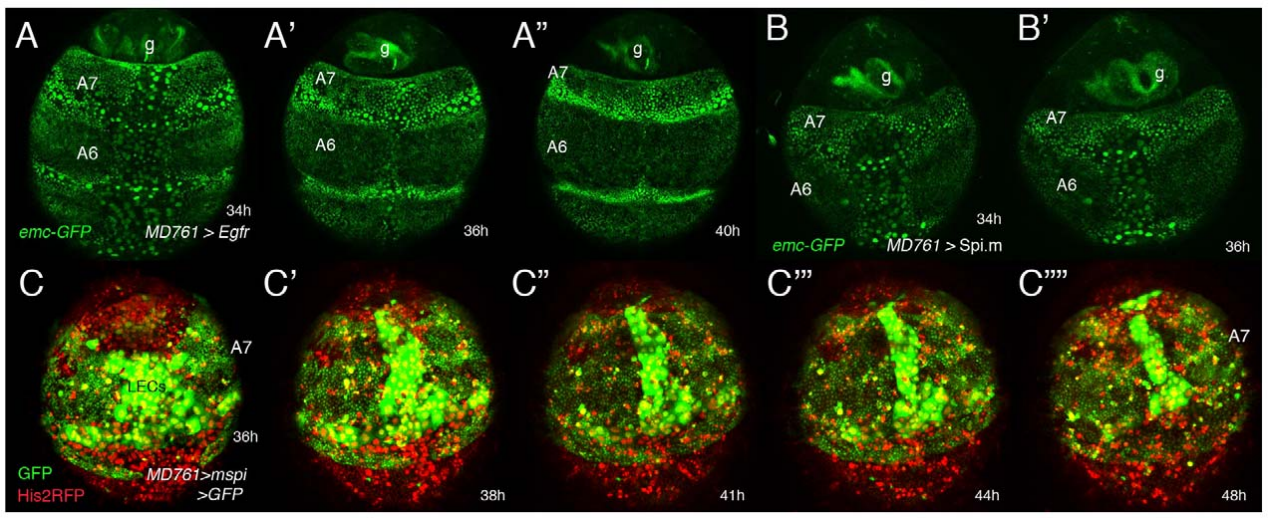
Genetic interactions between *emc* and the EGRF pathway. (A–B′) Stills from two videos (S14 and another one, not shown) done in pupae of the following genotypes: UAS-*Egfr*/+; *emc*-GFP *MD761*-Gal4/+ (A–A″) and *emc*-GFP *MD761*-Gal4/UAS-Spi.m-HRP (B, B′). The size of the A7 is increased in A–A″ and B, B′ with respect to the wildtype and *emc*-GFP expression seems slightly reduced in the A7 (compare with Figure 4E). (C–C″″) Snapshots from video S15 (∼35–48 h APF) showing a male pupa of the genotype UAS-Spi.m-GFP; *MD761*-Gal4 UAS-GFP/+. There is an excess of histoblasts in this segment (marked in green). The extrusion of LECs takes longer and that of histoblasts seems to be largely prevented. Numbers indicate approximate hours APF.

Both down-regulation of the EGFR pathway and increased *emc* expression seem to contribute to the suppression of the male A7 (Figure S3P–T). Overexpression of *spi* or *Egfr* shows mild effects in *emc*-GFP expression in the male A7 (video S14 and Figure 5A–B′; the wildtype in Figure 4E) but increases the histoblast number (Figure 1K, K′), so that the size of the A7 segment at about 36–44 h APF pupal stages is bigger than in the wildtype and many histoblasts are not extruded (Videos S14 and S15; Figure 5A–A″, B, B′, C–C″″). However, the detailed analysis of these movies suggest that, in addition to an increase in cell number, the strong activation of the EGFR pathway may also reduce extrusion, perhaps due to the slight effect observed in *emc*-GFP levels.

### Interactions between *extramacrochetae* and *wingless* in A7 development

A previous study [15] demonstrated that *wg* is expressed in the female, but not the male, A7 histoblasts, and that ectopic *wg* develops a small A7 in the male, partially pigmented and without bristles (Figure S4A). In *Abd-B* mutants there is ectopic *wg* in the male A7 [15], and this is important for the formation of the segment since the *Abd-B* mutant phenotype is partially rescued by diminishing *wg* activity (Figure S4B, compare with Figure 1H). To see if *emc* works in the A7 by regulating *wg* we looked to *wg* expression when Emc function is compromised. Wg antibody signal is not detected in the A7 of *MD761*-Gal4 UAS-GFP UAS-*emcRNAi* male pupae except, in some of them, for a very faint signal observed in some cells (Figure S4C). In the reciprocal experiment, however, we note a slight reduction in *emc*-GFP signal when *wg* expression is forced in the male A7 histoblasts (Figure S4D). This suggests that *emc* does not prevent A7 development by suppressing *wg* but that *wg* may regulate in part *emc* expression.

### A7 development in females

**Figure 6:**
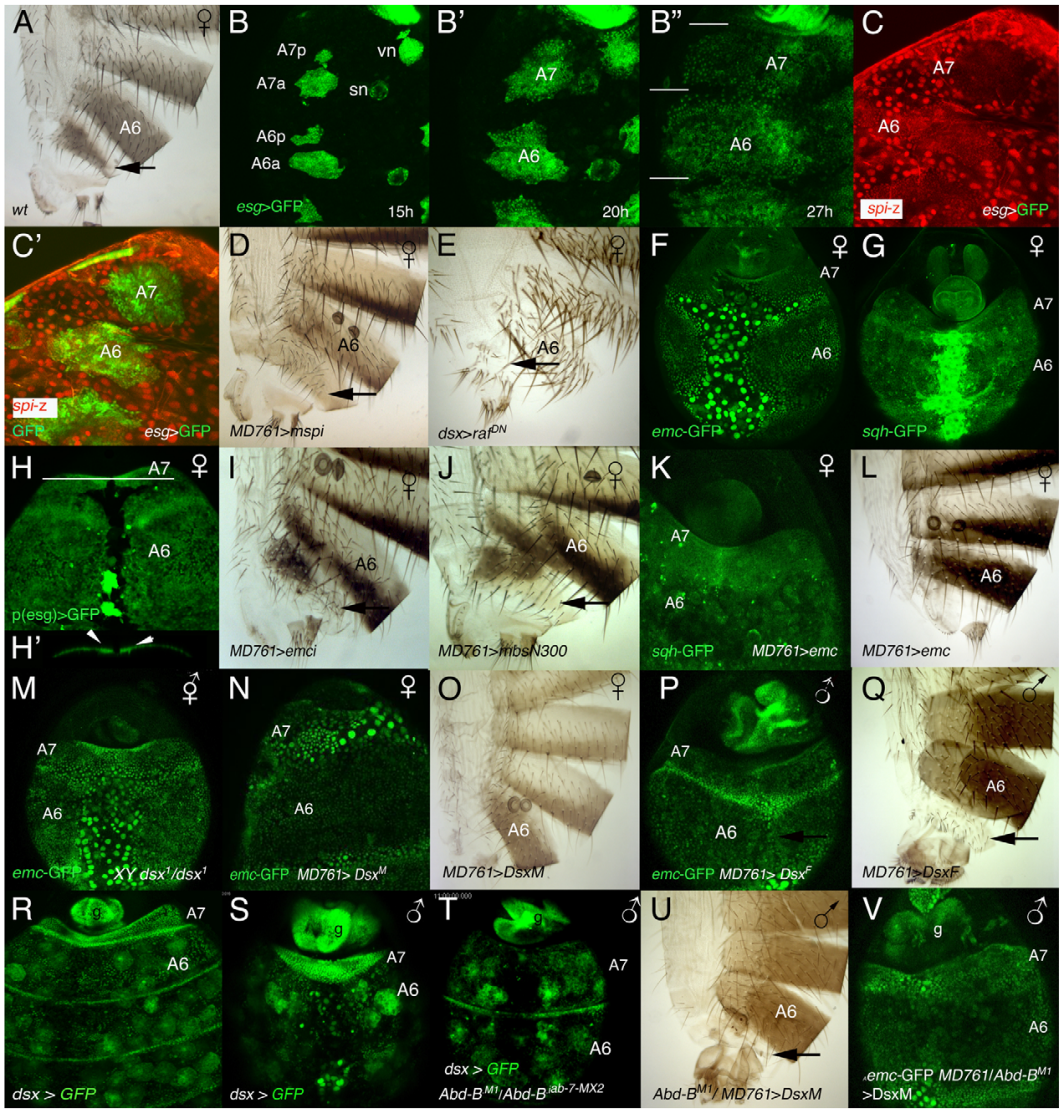
Relationship between sex determination and Hox information in the development of an A7. (A) Wildtype female adult, showing the small A7 segment (arrow). (B–B″) Stills taken from a movie in which the histoblasts of a ∼15–27 h APF (*p*)*esg*-Gal4 UAS-GFP female pupa are marked in green. Note that at the end of this period the A7 is slightly smaller than the A6 (segments separated by white lines). vn and sn indicate ventral nest and spiracular nests, respectively. Numbers indicate approximate hours of development APF. (C, C′). Posterior abdomen of an *esg*-Gal4 UAS-GFP/*spi*-lacZ female pupa from ∼25 h APF showing that the expression of *spi*-lacZ in the A7 (C, C′ in red) is reduced compared with that of the A6. Histoblasts are marked by *esg* expression in green (C′). (D) In UAS-Spi.m-GFP/+; *MD761*-Gal4/+ females the A7 is slightly bigger than in the wildtype (arrow; compare with A). (E) Posterior abdomen of a *dsx*-Gal4/UAS-Raf^DN^ female, in which the A7 segment is reduced. The larvae were grown at 25°C and transferred to 29°C at the third larval stage. (F, G) The expression of *emc*-GFP (F) and *sqh*-GFP (G) in the A7 of female pupae of about 36–38 h APF is not higher than in the A6 (compare with the male expression in Figs. 4E and 2F, respectively). (H) Posterior abdomen of a female pupa marked with (*p*)*esg*-Gal4 UAS-GFP at about 36 h APF. The optical section below (the white line indicates the plane of section) shows a slight accumulation of histoblasts in the central region of the segment under the epithelium (arrows in H′). This central dorsal region is partially absent in the adult female (see A). (I) Posterior abdomen of a UAS-*emcRNAi*/+; *MD761*-Gal4/+ female showing a small size increase in the dorsal region of the A7 (arrow: compare with Figure 6A). (J) Females expressing the MbsN300 protein in their A7 also show an enlarged dorsal domain (arrow). (K) In UAS-*emc*/+; *MD761*-Gal4/+ female pupae the expression of *sqh*-GFP is increased in the A7 (compare with G) and this segment disappears in the adult female (L). (M) X B^S^Y; *dsx^1^*/*emc*-GFP *dsx^1^* intersex pupa of ∼36 h APF, in which *emc* expression in the A7 is not up-regulated as in males. (N–Q) The expression of DsxM in the female A7 increases *emc*-GFP signal (N) and prevents the formation of the segment (O), whereas the expression of DsxF in the male A7 down-regulates *emc*-GFP expression (P) and develops an A7 (Q). (R, S) In female (R) or male (S) *dsx*-Gal4 UAS-GFP late pupae, the levels of *dsx* are higher in the A7 than in the A6. In the males, measurements show that the A7/A6 ratio in GFP signal intensity is 3,29±0,99 (n = 5). (T) In UAS-GFP/+; *dsx*-Gal4 *Abd-B^M1^*/*Abd-B^iab-7MX2^* male pupae, in which the A7 and A6 are transformed into A5, the levels of *dsx* in the A7 and A6 are similar. The round cells expressing *dsx*-Gal4 in R-T are probably fat cells [63]. (U) If the DsxM protein is expressed in an *Abd-B* mutant background (UAS-DsxM/+; *MD761*-Gal4/*Abd-B^M1^*), it almost completely rescues the transformation induced by the loss of *Abd-B* (compare with Figure 1C). 5 males of this genotype show this phenotype whereas 4 other males present a small A7 segment, sometimes in only one side. Cross made at 17°C. (V) In a male pupa of the UAS-DsxM/+; *emc*-GFP *MD761*-Gal4/*Abd-B^M1^* genotype the levels of *emc*-GFP expression in the A7 are high.

The wildtype female A7 is smaller than the A6 (Figure 6A). The initial stages of male and female pupal development are similar, including the reduction in cell division rate of histoblasts, although not so strong as in the male [15] (Figure 6B–B″), and the down-regulation of *spi* expression in the A7 (Figure 6C, C′). Consistently with a role of the EGFR pathway in controlling the A7 size, we observe that this size increases when we express Spi.m (Figure 6D) and it is reduced after the expression of a dominant negative form of the Raf protein (Figure 6E).

A significant difference, however, is seen at later stages. Contrary to what happens in males, the A7 levels of *emc*-GFP (Figure 6F) or *sqh*-GFP (Figure 6G) at about 35–40 h APF are similar to those observed in the A6, and although some histoblasts seem to be extruded in the central region of the segment (Figure 6H, H′), the massive effect occurring in males is not observed. However, *emc* mutant females present a slight but consistent increase in A7 size with respect to the wildtype (Figure 6I, compare with Figure 6A), perhaps due to the prevention of this extrusion. Consistent with this view, the A7 size increases in *MD761*-Gal4 UAS-*MbsN300* females (Figure 6J).

Our experiments indicate that *emc* levels are regulated by Dsx proteins in the A7 and that *emc*, in turn, regulates *sqh*: first, increasing *emc* in the female A7 elevates *sqh*-GFP levels and suppresses the A7 segment (Figure 6K, L); second, in XY *dsx^1^* intersexes, in which neither DsxF nor DsxM isoforms are made and which make a small A7 [40], the amount of *emc*-GFP in this segment at the time of extrusion seems lower than in the male A7 (Figure 6M); third, the expression of DsxM in the female A7 variably increases *emc*-GFP expression (Figure 6N) and suppresses the A7 (Figure 6O); finally, the expression of DsxF in the male A7 reduces *emc*-GFP signal (Figure 6P) and promotes the development of a segment (Figure 6Q). Nevertheless, high levels of *emc* are probably insufficient to determine the suppression of a segment: in *pnr*-Gal4 UAS-*emc* male pupae, in which *emc* expression is increased in the central dorsal region of the whole abdomen, the *sqh*-GFP signal is not elevated and there is no major extrusion of histoblasts in A6 or anterior segments (Video S16; Figure S5 and data not shown), suggesting that higher Emc levels than those obtained in this combination are required for extrusion and/or pointing to an *Abd-B*-dependent, *emc*-independent, contribution to delamination. Taken together, all these results suggest that changes in *emc* and *sqh* levels may mediate, at least in part, the activity of Dsx proteins to establish sexual dimorphism in the A7.

Recent results have shown that *dsx* is only expressed in specific cells throughout development, by and large those that will show sexually dimorphic characters [41]–[45]. To ascertain the expression of *dsx* in the posterior abdomen we have used *dsx*-Gal4 lines [44], [45] and found that the expression driven by these lines resembles that of *Abd-B*, with higher levels in the A7 of male or female pupae (Figure 6R, S). This suggested that *Abd-B* may regulate *dsx* expression and, according with this assumption, we found that down-regulation of *Abd-B* reduces *dsx* expression (Figure 6T). Similar results have been reported recently [46]. Our experiments suggest that changes in DsxF or DsxM levels in the A7, dictated by *Abd-B*, may mediate *Abd-B* effects. Consistently, expression of the DsxM protein in and *Abd-B* mutant background strongly reduces the A7 segment of males or females (Figure 6U, compare with Figure 1C, and data not shown) and substantially increases *emc*-GFP expression in males (Figure 6V). Pupae of this genotype show normal morphogenetic movements in the A7 (Video S17), suggesting the phenotypic rescue is not due to massive cell death.

## Discussion

The elimination of a part of an animal body is a major change occurring during morphogenesis and evolution. We have analyzed here the mechanisms required for one such change, the absence of the male seventh abdominal segment. Our study shows that the suppression of this segment involves the interplay between Hox and the sex determining genes, which regulate targets implementing the morphological change. The reduction or suppression of this segment is also a sexually dimorphic feature characteristic of higher Diptera, so the mechanisms shown here may be relevant for the evolution of morphology.

We have shown that in early pupa, during the second phase of cell division, there is a reduction in the number of A7 histoblasts, both in males and females ([15]; and this report), but stronger in males perhaps because *wg* is not expressed in the male A7 histoblasts [15]. It has been shown that fewer histoblasts result in a smaller adult segment [47]. Therefore, the reduced number of A7 histoblasts may account in part for the reduced size of the A7 segment in females. The control of the second phase of cell division involves the EGFR pathway [19], and we have found that *Abd-B* reduces the number of histoblasts in the A7 through down-regulation of EGFR activity. If we elevate this activity in the male A7 we observe an increase the number of histoblasts, that many of these cells remain at the surface at the time of extrusion and that a small A7 forms in the adult. It was also previously reported that a small A7 is observed in the male adult when expressing *vein*, an EGFR ligand [15]. It is possible that the high number of histoblasts obtained when over-expressing elements of the EGFR pathway makes many of them unable to be extruded by a “titration” effect, that is, there may be “too many” histoblasts for the invagination mechanism to extrude them at the correct time. However, the EGFR pathway may also hinder extrusion since we see lower levels of *emc*-GFP and also that many histoblasts remain at the surface after high EGFR activation.

At later pupal stages (around 35–40 h APF) there is the extrusion of the male A7 histoblasts. We have observed, however, that a few histoblasts also invaginate in the female A7, suggesting the male intensifies a mechanism present in both sexes. The extrusion requires the activity of *emc*, and correlates with higher *emc* expression in the male A7 histoblasts at about the time of extrusion. The invagination of histoblasts superficially resembles that of larval cells [12], and it also requires myosin activity. This would suggest that, due to the higher levels of *Abd-B* and DsxM, male A7 histoblasts may have adopted a mechanism similar to that used by LECs for their elimination. Recent reports [48], [49], however, suggest an alternative mechanism. In these manuscripts the authors demonstrate that an excess of proliferation in the epithelium leads to cell death-independent cell extrusion. Since we have observed that prevention of cell death in the male A7 does not cause the development of an A7 (although delamination is delayed), the mechanism driving extrusion may be more similar to that of an overproliferating epithelium than to that taking place in larval cells.

Our data are consistent with *emc* increasing the expression of *spaghetti-squash* to accomplish apical constriction and extrusion. However, high expression of *emc* may not be sufficient to effectively induce histoblast extrusion, suggesting other genes are required. Besides, a strong reduction of *emc* leads to a very small and poor differentiated male A7 segment (not shown), reflecting that this gene is required for several cellular functions, among them cell survival [50], [51]. Perhaps significantly, *emc* is also expressed in embryonic tissues preceding invagination of different structures in the embryo [52], suggesting a common requirement for invagination at different developmental stages. We think that *emc* forms part of complex networks that have, among other cellular functions, that of contributing to the extrusion of A7 histoblasts.

Although regulation of the EGFR pathway and *emc* are two key events in controlling male A7 development, previous experiments have also shown the contribution of the *wingless* gene, absent in male A7 but present in male A6 and female A7, in the development of this segment [15]. We have confirmed these results and also shown that a reduction in *wg* expression can partially suppress the *Abd-B* mutant phenotype. Absence of *wg* is probably required to reduce cell proliferation in the male A7 [15] but our data suggest *wg* may also be needed to maintain high *emc* levels. Apart from the role of *wg*, it was also shown that some A7a cells are transformed into A6p cells, thus reducing the number of A7 cells that might contribute to the adult segment [15]. Finally, the expression of *bric-a-brac* must also be down-regulated in male A7 histoblasts to eliminate this metamere [8]. Thus, this suppression is a complex process using different genes and mechanisms.

### Sex determination, Hox information and segment elimination

**Figure 7:**
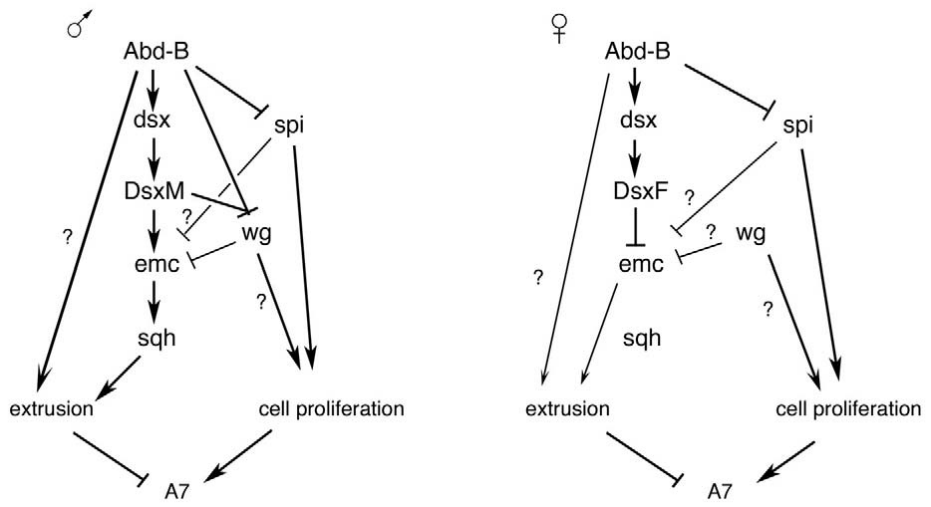
Schemes of genetic regulation in male and female A7. Summary of the results obtained together with those of Ref. 15. The different regulatory inputs take place in some cases at different times in development. See text for details.

The suppression of the male A7 depends ultimately on the levels of *Abd-B* expression. The role of this Hox gene is probably mediated in part by *dsx*, since *Abd-B* regulates *dsx* transcription ([46]; and this report) and *dsx* governs, in turn, the expression of genes required for cell proliferation and extrusion (Figure 7). That Hox genes regulate *dsx* expression has also been demonstrated in the male foreleg [53], suggesting that Hox genes specify the different parts of the body where sexual dimorphism may evolve. The different *dsx* isoforms (DsxF and DsxM) determine the outcome of this regulation. A significant difference between the activities of these two proteins in the A7 is the regulation of *emc* levels. In the female, *emc* expression is similar in the A7 and the A6 and, accordingly, histoblast extrusion in females is small and confined to the central dorsal region, a domain virtually absent in the adult tergite. By contrast, the DsxM isoform increases Emc expression to drive large extrusion of A7 cells and elimination of the segment (Figure 7).

Only the male A7, but not anterior abdominal segments, is eliminated. Therefore, the increase in *emc* expression, and subsequent events observed in the A7, depends on the higher *Abd-B* expression in the A7 in relation to the A6. Several Hox loci, like *Sex combs reduced*, *Ultrabithorax* or *Abd-B* are haplo-insufficient, and relatively small differences in the amount of some of these Hox proteins can drive major phenotypic changes [54]–[56], suggesting some downstream genes can sense these slight differences and implement major changes in morphology.

Previous studies have shown the cooperation of *Abd-B* and the sex determination pathway in controlling the pigmentation of the posterior abdomen [8], [9]. We think that *Abd-B* plays a dual role in regulating the morphology of the posterior abdomen. First, it regulates *dsx* expression, thus allowing the possibility to develop sexually dimorphic characters; second, it cooperates with Dsx proteins in establishing pattern (Figure 7). Part of the effect implemented by *Abd-B* may be mediated by the levels of expression of *dsx* (distinguishing male A6 from male A7), and from the nature of the Dsx proteins (male and female ones). Although there is no conclusive evidence that the different levels of *dsx* in the A6 and A7 play a role in development, we note that this difference correlates with that of *Abd-B* (and depends on it), that high levels of DsxM are sufficient to increase *emc*-GFP in the A7 of females and eliminate this segment, and that these same high levels similarly increase *emc*-GFP and partially rescue the *Abd-B* mutant phenotype in males. Hox genes, therefore, may provide a spatial cue along the anteroposterior axis to activate *dsx* transcription and allow the formation of sexually dimorphic characters, but they may also cooperate with Dsx proteins to determine different morphologies. This double control by Hox genes may apply to all the sexually dimorphic characters and be also a major force in evolution.

## Materials and Methods

### Genetics

We used the following mutations, P-lacZ, P-Gal4 and UAS lines, described in Flybase [57]: *Abd-B^M1^*, *Abd-B^M5^*, *Abd-B^Fab7-1^*, *Abd-B^iab-7MX2^*, *emc^1^*, *emc^P5C^*, *emc^FX199^* (*emc^9^*), *dsx^1^*, *spi*-lacZ (*spi^s3547^*), *aos*-lacZ (*aos^W11^*), *pnr*-Gal4, *en*-Gal4, *tsh*-Gal4 (*tsh^MD621^*), *esg*-Gal4 (*esg^NP5130^*); UAS-Spi.m-GFP, UAS-Spi.m-HRP, UAS-Ras^V12^, UAS-Raf^DN^, UAS-*Egfr*, UAS-*Egfr^DN^*, UAS-*aos*, UAS-*tra*, UAS-*emc*, UAS-*MbsN300*, UAS-*wg*, UAS-*Diap1*, UAS-*puc*, UAS-P35, UAS-GFP, UAS-RFP, UAS-dsRed, *sqh*-GFP, *zcl*-GFP (*zcl^2207^*), *nrg*-GFP (*nrg^G00305^*) and *hh*-Dsred (*hh^PyR215^*). Other constructs used are: *dsx*-Gal4 [44], [45], UAS-*nls-myc*-EGFP [58], UAS-*DsxF* and UAS-*DsxM* [41], UAS-Apoliner [59], *emc*-GFP (*emc^YB217^*) [39] and His2-RFP (His2Av-mRFP1) [60]. Permanent *esg*-Gal4 expression, referred to as (*p*)*esg*-Gal4 UAS-GFP was obtained in flies of the following genotype: *esg*-Gal4 *act*>*y^+^*>Gal4 UAS-GFP/CyO; UAS-*flp*/TM6B [12]. This combination allows marking histoblasts in late pupal stages, when *esg* expression fades away [12]. Stocks with the RNAi constructs were obtained from the Vienna Drosophila RNAi Center [61], the Transgenic RNAi Project at Harvard Medical School and the Genetic Resource Center (DGRC), Kyoto, Japan.

### Inverse PCR

Inverse PCR to analyze the MD761-Gal4 P-element insertion was performed as described (http://www.fruitfly.org/about/methods/index.html).

### Immunohistochemistry

The primary antibodies used are: mouse anti-Abd-B at a 1∶100 dilution ([62]; and Developmental Studies Hybridoma Bank, University of Iowa), and mouse and rabbit anti-ß-galactosidase at 1∶2000 (Cappel). Secondary antibodies were conjugated anti-mouse or anti-rabbit Fluor 488, 555 or 647 (Alexa) used at a 1∶200 dilution. Topro (TO-PRO-3; Molecular probes) was used to mark nuclei. Immunostaining and sample preparation were done according to standard methods. Pupal cuticle staining was performed as described [12] with small variations. White prepupa were transferred to empty vials and kept at 25°C for staging. Whole pupae were then bisected with a razor blade, cleaned with PBS and fixed for 90 minutes in 4% paraformaldehyde at 4°C, rinsed four times ×15 minutes in PBT-Triton (0.1% Triton X-100, 1% BSA in PBS) and blocked for at least 1 hour using PBT-BSA (1% bovine serum albumin (BSA) in PBT). Antibodies treatment and mounting were done following standard procedures.

### *In vivo* imaging and image analysis

Leica TCS SPE and Zeiss LSM700 confocal microscopes were used to capture both still images and time-lapse movies. All the confocal images are maximum intensity projections. Staging of the pupae was performed as described [12]. APF stands for hours after puparium formation, taking the eversion of anterior spiracles in white prepupae as a reference. Male (XY) pupae of the genotype X *B^S^*Y *dsx^1^*/*dsx^1^* were distinguished from the XX siblings by the *B^S^* mutation. The use of different setting conditions in the capturing the different movies makes to see the rotation of the genitalia look normal (clockwise) or inverted (anti-clockwise). All the movies were captured at 10 minutes intervals keeping the laser intensity at a minimum to avoid damaging the pupae. Unless specified, all the images correspond to z-stacks with slides taken at an optimum distance to get the whole structure 3D reconstruction of z-stacks, and mounting of time-lapse movies in AVI format was performed with Leica Confocal Software (LAS AF Lite) or Zeiss ZEN2009 software. ImageJ (NIH Image) and Photoshop 7.0 (Adobe Corporation) were used for data processing, cell counting and measurement of signal intensity.

### Adult cuticle preparations

Flies were kept in a mixture of ethanol: glycerol (3∶1), dissected, macerated in 10% KOH-at 90°C for three minutes, washed with PBT (1% Triton X-100 in PBS), rinsed 3×15 minutes in PBS and mounted in Glycerol for inspection under a compound microscope.

## Supporting Information

Figure S1 **Effect of** *Abd-B* **and sex determination mutations and development of the male A7.** (A) Wildtype male abdomen. (B) Wildtype female abdomen. Note the A7 segment (arrow), absent in the male. (C) *Abd-B* mutant male (*Abd-B^M1^*/*Abd-B^M5^*). See the big A7 segment (arrow). A very similar phenotype is also observed in females of this genotype. (D) *tsh*-Gal4 UAS-*tra* chromosomal (XY) male, in which the abdomen is transformed into a female due to the expression of *tra*. See the formation of an A7 segment (arrow). (E–E″) *nrg*–GFP pupa (*neuroglian*-GFP marks cell membranes and it is used to see cell size) of approximately 24 h APF, in which it is observed that the A7 histoblasts are of bigger size than the A6 ones. Insets show details for A6 (E′) and A7 histoblast nests (E″). (F–H′). Male pupae of about 25 h APF, marked with anti-Abd-B (in red) and Topro (in blue), of the following genotypes: *MD761*-Gal4/*Abd-B^M1^* (F, F′), wild-type (G, G′) and *Abd-B^Fab7-1^* (H, H′). Abd-B expression is higher in the wild-type A7 than in the A6 (the A7/A6 ratio in Abd-B signal is 2,14±0,22, n = 5), and this correlates with A7 histoblast nests having less number of histoblasts and their being bigger. In the mutant combinations that transform A7 into A6 (*MD761*-Gal4/*Abd-B^M1^*) or A6 into A7 (*Abd-B^Fab7-1^*), these morphological characteristics change according to the *Abd-B* levels. (I, I′) Posterior part of an *esg*-Gal4 UAS-GFP/+; *aos*-lacZ/+ pupa, in which the A5 and A6 anterior histoblast nests, marked by GFP (green in I), show low *aos* expression (in white in I′, in red in I, arrowheads; the A6 anterior nest show a fold in the cuticle) whereas in the A5p, A6p (black arrows) and A7 nests (white arrow) the *aos* signal is almost absent. LECs, larval epidermal cells. (J) Posterior abdomen of a UAS-Spi.m-GFP; *tub*-Gal80^ts^/+; *dsx*-Gal4/+ male, transferred from 25 to 29°C during the third larval instar. A relatively big A7 segment develops (see also Figure 1F). (K) A similar A7 segment develops in males of the *MD761*-Gal4/UAS-Ras^V12^ genotype. (L, M) The big A7 segment of UAS-*Abd-BRNAi*/+; *MD761*-Gal4 UAS-*y^+^*/+males (Figure 1H) is reduced if we also express the dominant-negative form of the Epidermal growth factor receptor (UAS-Egfr^DN^/+; *MD761*-Gal4/*UAS-Abd-BRNAi*, L) or the Argos (UAS-*aos*/+; *MD761*-Gal4/*UAS-Abd-BRNAi*, M) proteins. Arrows in J-M mark the A7.
(TIF)

Figure S2 **Extrusion of A7 histoblasts.** (A) Snapshots from video S4 (from about 32 to 41 h APF), in which the histoblasts are marked with (*p*)*esg*-Gal4 UAS-GFP, showing the progressive elimination of A7 cells as left and right histoblast nests meet. (B, C) Stills from movies S7 and S8, corresponding to His2A-RFP/+; *MD761*-Gal4 UAS-MbsN300 UAS-GFP (B) and His2A-RFP/+; *MD761*-Gal4 UAS-GFP (C) ∼34–40 h APF male pupae. See that in the wildtype at about 40 h APF both LECs (of a bigger size) and histoblasts (smaller size) from the A7, marked in green, have been extruded, whereas in the mutant phenotype some LECs and most histoblasts persist in the surface (arrowhead). Hours indicate the approximate time APF. (D, E) Adults expressing the MbsN300 construct show a small A7, without pigmentation or bristles (E); compare with the wildtype, without A7, in D.
(TIF)

Figure S3 **Role of** *emc* **in suppressing the male A7 and interactions between** *emc* **and** *Abd-B* **, or** *emc* **and the EGFR pathway.** (A–C) A reduction in *emc* levels obtained in *emc^1^*/*emc^P5C^* (A), *emc^FX119^*/*emc^P5C^* (B), or expressing an *emc*RNAi construct at 17°C (UAS-*emcRNAi*/+; *MD761*-Gal4/+, C), produces a small A7 segment in males. In some *emc* mutant males there are occasionally one or more bristles in the sixth sternite, perhaps due to the regulation by *emc* of bristle development. No *emc* mutation shows even a tiny A7 in heterozygous condition. (D–G) In *Abd-B^M1^*/*emc^FX119^* (E), *Abd-B^M1^*/*emc^P5C^* (F) or *emc^P5C^ Abd-B^M1^*/+ (G) males, the size of the male A7 is significantly bigger than that observed in *Abd-B^M1^*/+ males (D). (H–J) Similarly, in *Abd-B^M5^*/+ males (H), the A7 is smaller than in *Abd-B^M5^*/*emc^P5C^* (I) or *Abd-B^M5^*/*emc^FX119^* (J) adults. Comparable interactions are observed with the *MD761*-Gal4 line: in *MD761*-Gal4/+ males there is no A7 or, with low penetrance, a very tiny piece of cuticle (K; see also Figure 1G); by contrast, a bigger segment is seen in *emc^P5C^ MD761-Gal4/+* (L) or *MD761*-Gal4/*emc^P5C^* (M) males. (N) In *MD761*-Gal4/*Abd-B^M1^* adults there is an almost complete transformation of the A7 into the A6, but in UAS-*emc*/+; *MD761*-Gal4/*Abd-B^M1^* males the size of the transformed A7 is significantly reduced (O). (P–T) Adults males of the following genotypes: *emc^P5C^*/*MD761*-Gal4 (P), *emc^P5C^*/*emc^P5C^ MD761*-Gal4 (Q), UAS- Ras^V12^/+; *MD761*-Gal4/+ (R), UAS- Ras^V12^/+; *emc^P5C^*/*MD761*-Gal4 (S) and UAS- Ras^V12^/+; *emc^P5C^*/*emc^P5C^ MD761*-Gal4 (T). Note that the combination of *emc* mutations and EGFR activity increases the size of the male A7 segment. In the UAS-Ras^V12^/+; *emc^P5C^*/*emc^P5C^ MD761*-Gal4 genetic combination the phenotype is variable. Arrows indicate the A7 segment.
(TIF)

Figure S4 **Relationship between** *wingless* **and** *extramacrochetae* **in male A7 development.** (A) *MD761*-Gal4 UAS-*y^+^*/UAS-*wg* male. A small A7 is observed, without bristles and partially pigmented (arrow). (B). In UAS-*wgRNAi*/+; *MD761*-Gal4/UAS-*Abd-BRNAi* the transformation of the A7 into A6 caused by the loss of *Abd-B* is partially suppressed by the concomitant reduction of *wg* (compare with Figure 1H). (C). The reduction of *emc* expression (*emc^P5C^/emc^P5C^* male pupa) does not activate *wg* expression in the A7. Only a very weak signal is observed in some cases (arrow). (D) The ectopic expression of *wg* in the A7 of a ∼38 h APF *emc*-GFP MD761/UAS-*wg* male pupa reduces *emc*-GFP expression in some cells of this segment (compare with Figure 4E); g, genitalia.
(TIF)

Figure S5 **Over-expression of** *emc* **does not induce massive extrusion of histoblasts in A6 or anterior segments.** (A) Snapshots from video S16 (*pannier*-Gal4 UAS-*emc* UAS-GFP male) of about 36–50 h APF, showing that histoblasts of segments anterior to the A7 do not show major extrusion.
(TIF)

Video S1 **Histoblast nest growth in male pupae.** The movie shows how A7 and A6 histoblast nests of an *esg*-Gal4 UAS-*nls-myc*-GFP pupa grow during the 15–27 h APF period. The final outcome is that the A7a and A7p nests (now fused) are reduced in cell number with respect to that of anterior nests. See also Figure 1A–A″.
(AVI)

Video S2 **Elimination of A8 and A7 segments in male pupae.** Male pupa of about 25–34 h APF, in which posterior compartments are marked with *en*-Gal4 UAS-GFP (in green), and nuclei with His2A-RFP (in red), showing the progressive elimination of the A8 (at the top and close to the genitalia, which rotates) and then of the A7. It was previously shown [15] that there is *de novo en* expression in A6p of cells originally belonging to A7a. This transformation may contribute to the apparent reduction of the A7 segment. Histoblasts are difficult to see under the great number of moving macrophages or hemocytes. Larval cells are distinguished by their bigger size. See also Figure 2A–A′″.
(AVI)

Video S3 **Elimination of A8 and A7 segments in male pupae (II).** This movie continues the previous one, from about 36 h to 50 h APF, until the A7 is eliminated and A6p contacts the genitalia (which ends its rotation). See also Figure 2B–B′″.
(AVI)

Video S4 **Elimination of male A7 cells (I).** The elimination of A7 cells is observed in a (*p*)*esg*-Gal4 UAS-GFP male pupa from ∼32–42 h APF. The histoblasts are marked in green. Note how left and right histoblast nests fuse and how the A7 segment (at the top) gets reduced in later stages. See also Figure S2A.
(AVI)

Video S5 **Elimination of male A7 cells (II).** In this movie, spanning from ∼35–50 h APF, the male pupa is marked with *nrg*-GFP, which labels cell membranes of larval cells (big ones) and histoblasts. See that the A7 and A6 nests are still separated by a few larval cells at the beginning of the video. Note also how bristle precursors from the A6 move from the lower part of the video to the top as the A7 cells are extruded. The genitalia rotate at the top of the video. See also Figure 2C–C′″.
(AVI)

Video S6 **Elimination of male A6 and A7 cells in** *Abd-B^Fab7-1^* **mutants.** Movie from ∼36–50 h APF, in which posterior compartments are marked with *hh*-RFP (in red) and cell membranes with *zcl22*-GFP (in green). See how in the *Abd-B^Fab7-1^* homozygous male pupa both the A6 and A7 disappear while the A5p moves posteriorly to contact the genitalia (at the top). See also Figure 2E–E″.
(AVI)

Video S7 **The inhibition of myosin II activity prevents extrusion of larval cells and histoblasts.** Movie from ∼34–40 h APF. The male is expressing MbsN300 and GFP in the A7 (His2A-RFP/+; *MD761*-Gal4 UAS-GFP/UAS-MbsN300 pupa). Compare the delayed extrusion of LECs (big size) and the almost absence of histoblast invagination with the extrusion observed in the following movie. The A7 is marked in green and the nuclei in red. See also Figure S2B.
(AVI)

Video S8 **Disappearance of the A7 segment in His2A-RFP** *MD761* **-Gal4 UAS-GFP males.** The timing of the movie is like that of the previous one. Note that, different from it, larval cells and histoblasts are being extruded. His2A-RFP, marking nuclei, is in red, and the A7 segment is labeled in green. Note the rotation of the genitalia. See also Figure S2C.
(AVI)

Video S9 **Delamination of histoblasts (I).** We show a movie from about 38–44 h APF in a (*p*)*esg*-Gal4 UAS-GFP male pupa. Left and right histoblast nests from the A7 meet at the dorsal midline and at the same time the histoblasts delaminate. See also Figure 3A–A″″.
(AVI)

Video S10 **Delamination of histoblasts (II).** The movie, from ∼42 to 50 h APF, represents the delamination of A7 histoblasts, marked in green in a (*p*)*esg*-Gal4 UAS-GFP male pupa. Note that at the beginning of the movie the width of the epithelium in the A7 region is bigger than at the end of the movie (after delamination). Unlike in the rest of the movies, *Z* stacks were collected every 5 min. instead of 10 min. to better visualize the delamination of cells. See also Figure 3B–B″″.
(AVI)

Video S11 **Extrusion of A7 histoblasts when cell death is prevented.** The video shows the extrusion of A7 histoblasts in a male pupa of about 36–42 h APF of the genotype *esg*-Gal4 *act*>*y^+^*>Gal4/UAS-*Diap1*; UAS-*flp*/UAS-*Diap1*. See also Figure 3E–E′″.
(AVI)

Video S12 **Elimination of the A7 in wildtype males.** A ∼36–48 h APF *en*-Gal4 UAS-GFP/His2A-RFP male pupa, in which posterior compartments are marked in green and nuclei are marked in red, showing the extrusion of A7 and how the A6p band ends up contacting with the genitalia. See also Figure 4I–I″.
(AVI)

Video S13 **The A7 is not eliminated in** *emc* **mutant males.** Evolution from ∼36 to ∼48 h APF of an *en*-Gal4 UAS-GFP/His2A-RFP; *emc^P5C^*/*emc^P5C^* male pupa, in which posterior compartments are marked in green and nuclei in red, showing that the A7 cells are not extruded and the A6p does not extend posteriorly to contact the genitalia (compare with Video S12). See also Figure 4J–J″.
(AVI)

Video S14 **The augmented expression of Egfr increases the number of histoblasts and the A7 size.** The male pupa has the genotype UAS-*Egfr/+*; *emc*-GFP *MD761*-Gal4/+ and goes from ∼35 to 44 h APF. See that an A7 remains after all the larval cells have been eliminated. See also Figure 5A–A″.
(AVI)

Video S15 **Increased expression of Spi.m prevents the extrusion of many histoblasts in the male A7.** Male pupa of ∼35–48 h APF. The genotype is UAS-Spi.m-GFP; *MD761*-Gal4 UAS-GFP/+. See the large number of histoblasts. The larval cells (big size) take longer to be extruded but almost all finally do so, whereas most histoblasts remain at the surface. See also Figure 5C–C″″.
(AVI)

Video S16 **Ectopic** *emc* **is not sufficient to extrude histoblasts of segments anterior to the A7.** The movie has been obtained in a UAS-*emc*/UAS-GFP; *pnr*-Gal4/+ male of ∼36–50 h APF. See that the histoblasts of the A6 and A5 segments remain at the surface. See also Figure S5.
(AVI)

Video S17 **The expression of DsxM increases** *emc* **-GFP expression in an** *Abd-B* **mutant background.** The genotype of the male pupa is UAS-DsxM/+; *emc*-GFP *MD761*-Gal4/*Abd-B^M1^*. There is increase of *emc*-GFP signal in the A7 even though the pupa is mutant for *Abd-B*. See normal cell divisions and cell movements. See also Figure 6V.
(AVI)
