# Supplementary figures and images for: Drosophila Hox and Sex-Determination Genes Control Segment Elimination through EGFR and extramacrochetae Activity
Source: PLoS Genet. 2012 Aug 9;8(8):e1002874. doi: 10.1371/journal.pgen.1002874 (PMC3415437; doi:10.1371/journal.pgen.1002874)

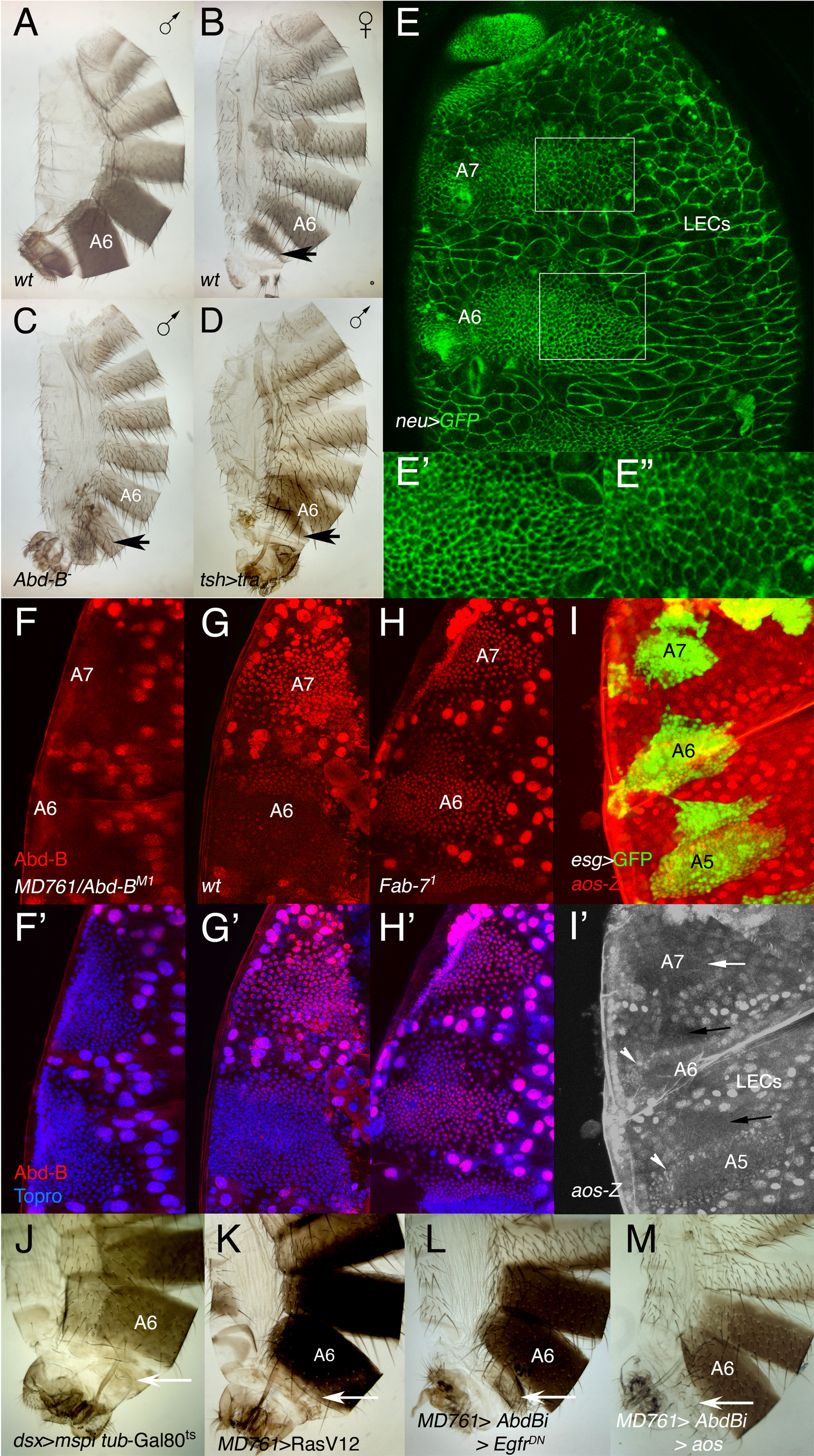

Supplement: Figure S1 — Effect of Abd-B and sex determination mutations and development of the male A7. (A) Wildtype male abdomen. (B) Wildtype female abdomen. Note the A7 segment (arrow), absent in the male. (C) Abd-B mutant male (Abd-BM1/Abd-BM5). See the big A7 segment (arrow). A very similar phenotype is also observed in females of this genotype. (D) tsh-Gal4 UAS-tra chromosomal (XY) male, in which the abdomen is transformed into a female due to the expression of tra. See the formation of an A7 segment (arrow). (E–E″) nrg–GFP pupa (neuroglian-GFP marks cell membranes and it is used to see cell size) of approximately 24 h APF, in which it is observed that the A7 histoblasts are of bigger size than the A6 ones. Insets show details for A6 (E′) and A7 histoblast nests (E″). (F–H′). Male pupae of about 25 h APF, marked with anti-Abd-B (in red) and Topro (in blue), of the following genotypes: MD761-Gal4/Abd-BM1 (F, F′), wild-type (G, G′) and Abd-BFab7-1 (H, H′). Abd-B expression is higher in the wild-type A7 than in the A6 (the A7/A6 ratio in Abd-B signal is 2,14±0,22, n = 5), and this correlates with A7 histoblast nests having less number of histoblasts and their being bigger. In the mutant combinations that transform A7 into A6 (MD761-Gal4/Abd-BM1) or A6 into A7 (Abd-BFab7-1), these morphological characteristics change according to the Abd-B levels. (I, I′) Posterior part of an esg-Gal4 UAS-GFP/+; aos-lacZ/+ pupa, in which the A5 and A6 anterior histoblast nests, marked by GFP (green in I), show low aos expression (in white in I′, in red in I, arrowheads; the A6 anterior nest show a fold in the cuticle) whereas in the A5p, A6p (black arrows) and A7 nests (white arrow) the aos signal is almost absent. LECs, larval epidermal cells. (J) Posterior abdomen of a UAS-Spi.m-GFP; tub-Gal80ts/+; dsx-Gal4/+ male, transferred from 25 to 29°C during the third larval instar. A relatively big A7 segment develops (see also Figure 1F). (K) A similar A7 segment develops in males of the MD761-Gal4/UAS-RasV12 [file pgen.1002874.s001.tif]

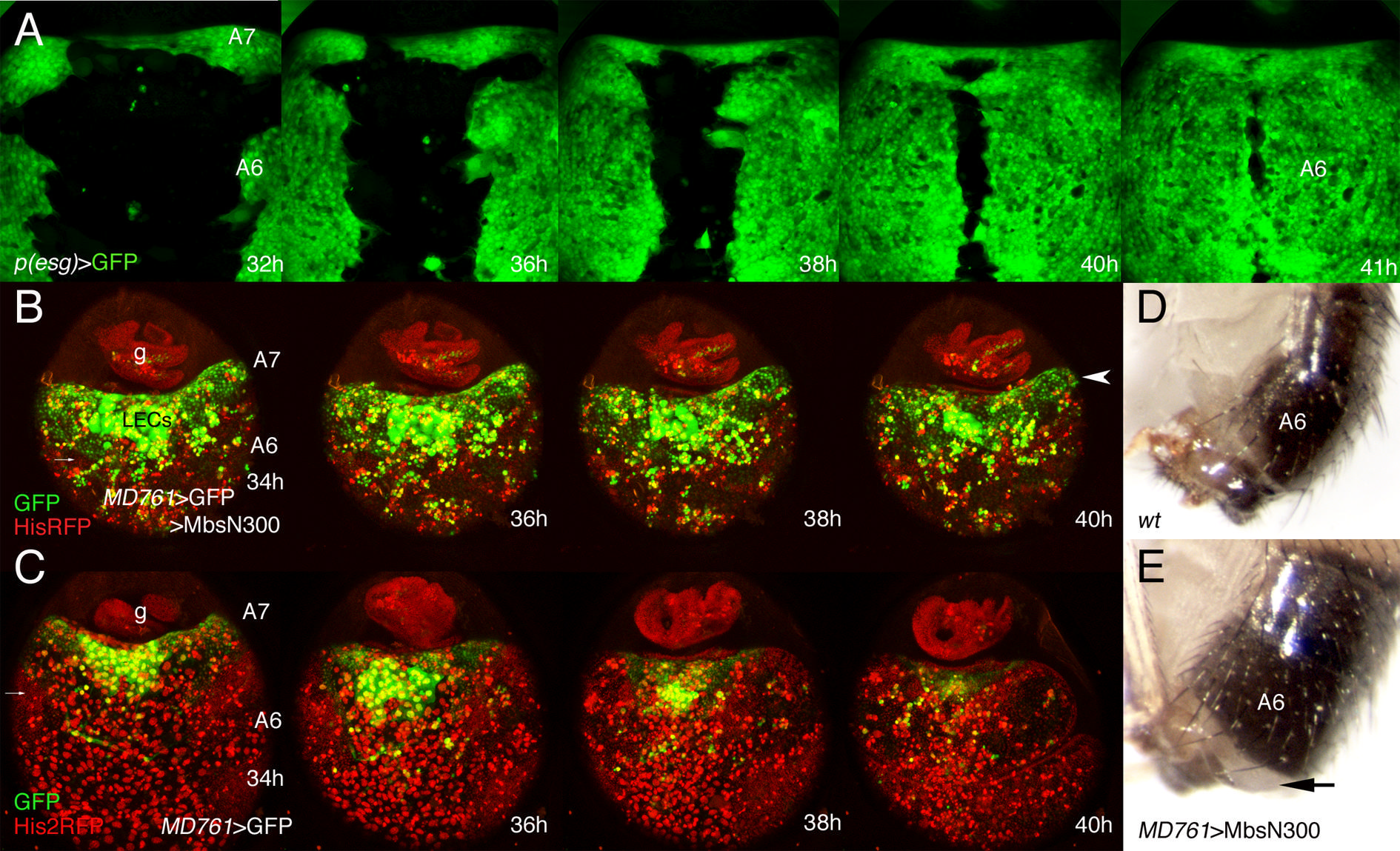

Supplement: Figure S2 — Extrusion of A7 histoblasts. (A) Snapshots from video S4 (from about 32 to 41 h APF), in which the histoblasts are marked with (p)esg-Gal4 UAS-GFP, showing the progressive elimination of A7 cells as left and right histoblast nests meet. (B, C) Stills from movies S7 and S8, corresponding to His2A-RFP/+; MD761-Gal4 UAS-MbsN300 UAS-GFP (B) and His2A-RFP/+; MD761-Gal4 UAS-GFP (C) ∼34–40 h APF male pupae. See that in the wildtype at about 40 h APF both LECs (of a bigger size) and histoblasts (smaller size) from the A7, marked in green, have been extruded, whereas in the mutant phenotype some LECs and most histoblasts persist in the surface (arrowhead). Hours indicate the approximate time APF. (D, E) Adults expressing the MbsN300 construct show a small A7, without pigmentation or bristles (E); compare with the wildtype, without A7, in D. (TIF) [file pgen.1002874.s002.tif]

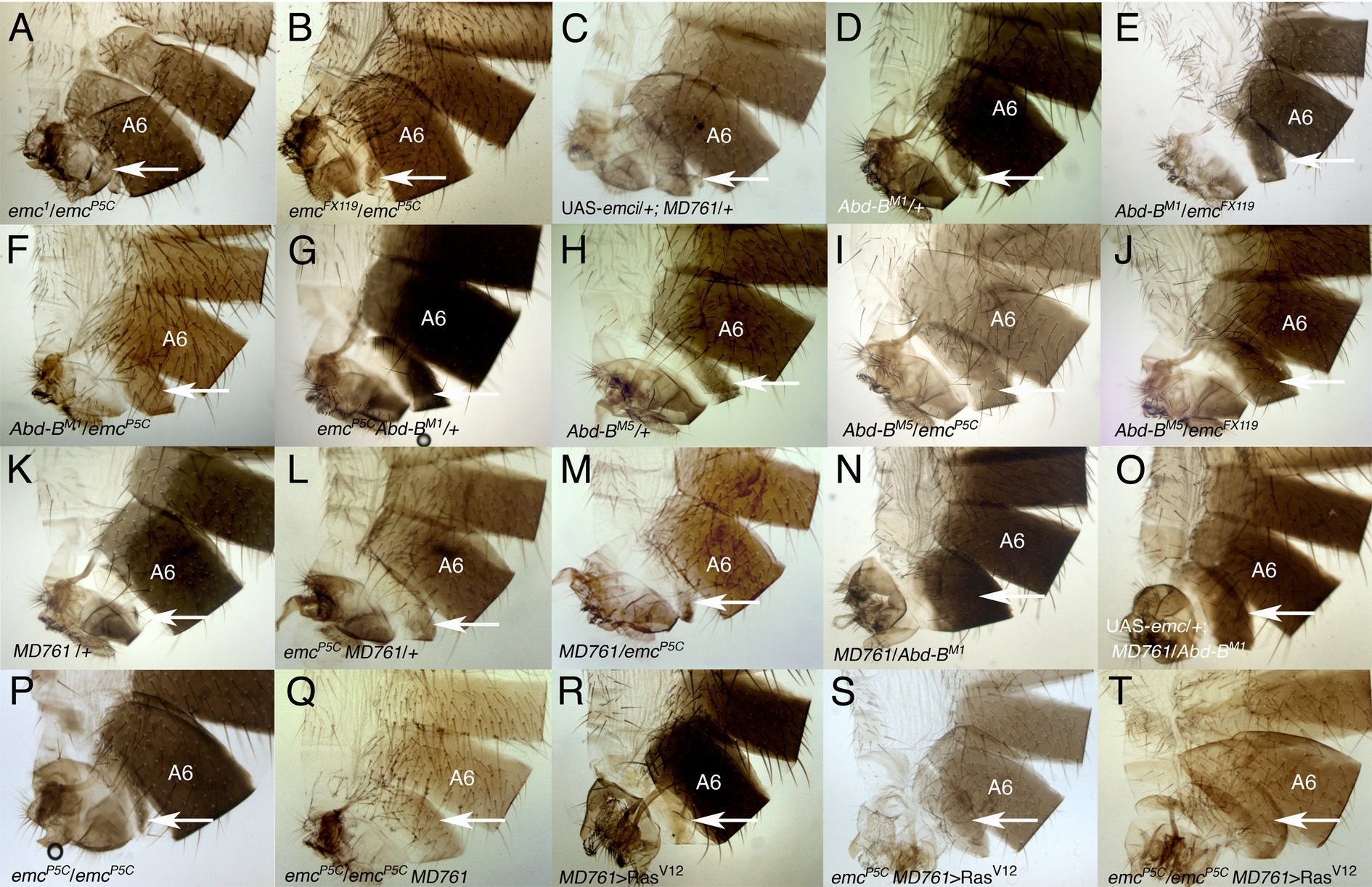

Supplement: Figure S3 — Role of emc in suppressing the male A7 and interactions between emc and Abd-B , or emc and the EGFR pathway. (A–C) A reduction in emc levels obtained in emc1/emcP5C (A), emcFX119/emcP5C (B), or expressing an emcRNAi construct at 17°C (UAS-emcRNAi/+; MD761-Gal4/+, C), produces a small A7 segment in males. In some emc mutant males there are occasionally one or more bristles in the sixth sternite, perhaps due to the regulation by emc of bristle development. No emc mutation shows even a tiny A7 in heterozygous condition. (D–G) In Abd-BM1/emcFX119 (E), Abd-BM1/emcP5C (F) or emcP5C Abd-BM1/+ (G) males, the size of the male A7 is significantly bigger than that observed in Abd-BM1/+ males (D). (H–J) Similarly, in Abd-BM5/+ males (H), the A7 is smaller than in Abd-BM5/emcP5C (I) or Abd-BM5/emcFX119 (J) adults. Comparable interactions are observed with the MD761-Gal4 line: in MD761-Gal4/+ males there is no A7 or, with low penetrance, a very tiny piece of cuticle (K; see also Figure 1G); by contrast, a bigger segment is seen in emcP5C MD761-Gal4/+ (L) or MD761-Gal4/emcP5C (M) males. (N) In MD761-Gal4/Abd-BM1 adults there is an almost complete transformation of the A7 into the A6, but in UAS-emc/+; MD761-Gal4/Abd-BM1 males the size of the transformed A7 is significantly reduced (O). (P–T) Adults males of the following genotypes: emcP5C/MD761-Gal4 (P), emcP5C/emcP5C MD761-Gal4 (Q), UAS- RasV12/+; MD761-Gal4/+ (R), UAS- RasV12/+; emcP5C/MD761-Gal4 (S) and UAS- RasV12/+; emcP5C/emcP5C MD761-Gal4 (T). Note that the combination of emc mutations and EGFR activity increases the size of the male A7 segment. In the UAS-RasV12/+; emcP5C/emcP5C MD761-Gal4 genetic combination the phenotype is variable. Arrows indicate the A7 segment. (TIF) [file pgen.1002874.s003.tif]

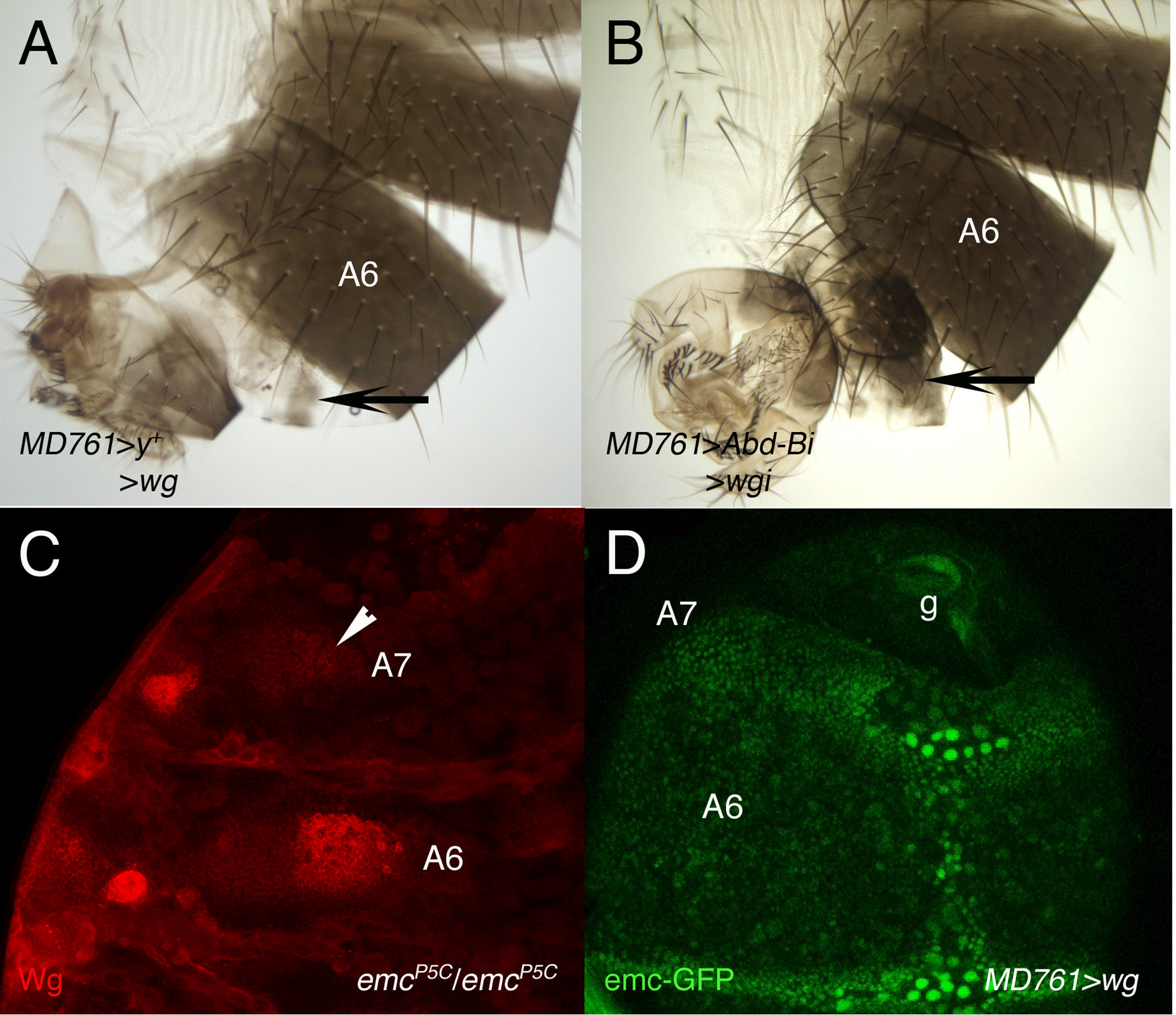

Supplement: Figure S4 — Relationship between wingless and extramacrochetae in male A7 development. (A) MD761-Gal4 UAS-y+/UAS-wg male. A small A7 is observed, without bristles and partially pigmented (arrow). (B). In UAS-wgRNAi/+; MD761-Gal4/UAS-Abd-BRNAi the transformation of the A7 into A6 caused by the loss of Abd-B is partially suppressed by the concomitant reduction of wg (compare with Figure 1H). (C). The reduction of emc expression (emcP5C/emcP5C male pupa) does not activate wg expression in the A7. Only a very weak signal is observed in some cases (arrow). (D) The ectopic expression of wg in the A7 of a ∼38 h APF emc-GFP MD761/UAS-wg male pupa reduces emc-GFP expression in some cells of this segment (compare with Figure 4E); g, genitalia. (TIF) [file pgen.1002874.s004.tif]

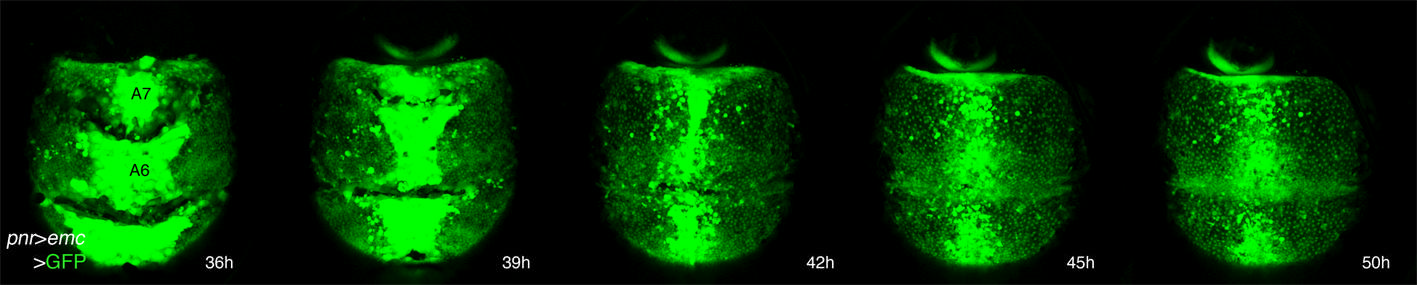

Supplement: Figure S5 — Over-expression of emc does not induce massive extrusion of histoblasts in A6 or anterior segments. (A) Snapshots from video S16 (pannier-Gal4 UAS-emc UAS-GFP male) of about 36–50 h APF, showing that histoblasts of segments anterior to the A7 do not show major extrusion. (TIF) [file pgen.1002874.s005.tif]
